# REDHORSE-REcombination and Double crossover detection in Haploid Organisms using next-geneRation SEquencing data

**DOI:** 10.1186/s12864-015-1309-7

**Published:** 2015-02-26

**Authors:** Jahangheer S Shaik, Asis Khan, Stephen M Beverley, L David Sibley

**Affiliations:** Department of Molecular Biology, Washington University School of Medicine, Box 8230, , 600 S. Euclid Ave., St. Louis, MO 63110 USA; Present address: Molecular Parasitology Unit, Laboratory for Parasitic Diseases, NIAID, NIH, Bethesda, MD 20892 USA

**Keywords:** Next-generation sequencing, Recombination detection, Conventional crossovers, Double crossovers, Haploid genome, Toxoplasma gondii, Multiple sequence alignments, Single nucleotide variations, Merged allele file and allele extraction

## Abstract

**Background:**

Next-generation sequencing technology provides a means to study genetic exchange at a higher resolution than was possible using earlier technologies. However, this improvement presents challenges as the alignments of next generation sequence data to a reference genome cannot be directly used as input to existing detection algorithms, which instead typically use multiple sequence alignments as input. We therefore designed a software suite called REDHORSE that uses genomic alignments, extracts genetic markers, and generates multiple sequence alignments that can be used as input to existing recombination detection algorithms. In addition, REDHORSE implements a custom recombination detection algorithm that makes use of sequence information and genomic positions to accurately detect crossovers. REDHORSE is a portable and platform independent suite that provides efficient analysis of genetic crosses based on Next-generation sequencing data.

**Results:**

We demonstrated the utility of REDHORSE using simulated data and real Next-generation sequencing data. The simulated dataset mimicked recombination between two known haploid parental strains and allowed comparison of detected break points against known true break points to assess performance of recombination detection algorithms. A newly generated NGS dataset from a genetic cross of *Toxoplasma gondii* allowed us to demonstrate our pipeline. REDHORSE successfully extracted the relevant genetic markers and was able to transform the read alignments from NGS to the genome to generate multiple sequence alignments. Recombination detection algorithm in REDHORSE was able to detect conventional crossovers and double crossovers typically associated with gene conversions whilst filtering out artifacts that might have been introduced during sequencing or alignment. REDHORSE outperformed other commonly used recombination detection algorithms in finding conventional crossovers. In addition, REDHORSE was the only algorithm that was able to detect double crossovers.

**Conclusion:**

REDHORSE is an efficient analytical pipeline that serves as a bridge between genomic alignments and existing recombination detection algorithms. Moreover, REDHORSE is equipped with a recombination detection algorithm specifically designed for Next-generation sequencing data. REDHORSE is portable, platform independent Java based utility that provides efficient analysis of genetic crosses based on Next-generation sequencing data. REDHORSE is available at http://redhorse.sourceforge.net/.

**Electronic supplementary material:**

The online version of this article (doi:10.1186/s12864-015-1309-7) contains supplementary material, which is available to authorized users.

## Background

Next-generation sequencing (NGS) provides a high-resolution snapshot of the sequenced genomes. The end product of sequencing is short nucleotide fragments called “reads” that are routinely aligned to the genome to generate alignment maps. These alignments cannot be directly used as input to the existing recombination detection (RD) algorithms [1-5] that instead use multiple sequence alignments (MSA) as input. Also, the existing RD algorithms cannot handle the large volume of data generated using NGS platforms due to memory limitations. There are currently no flexible analytical pipelines to analyze NGS datasets and to extract genetic markers, define allele composition, construct contigs, and generate MSAs. There is therefore a need for utilities and an efficient pipeline to summarize the data systematically [6]. The primary purpose of REDHORSE is to extract genetic markers from parental genomes and to generate MSAs from recombinant progeny so that these data can be used as input to existing RD algorithms. REDHORSE therefore serves as a bridge to reduce the distance between outputs from NGS platforms and the specific requirements of the RD algorithms to facilitate their applicability. In addition, REDHORSE is equipped with a RD algorithm that is specially designed for NGS data. The RD algorithm in REDHORSE extracts recombinations from NGS datasets using not only the contigs but also using genomic positions of the markers that were used to build the contigs.

Prior to developing our RD algorithm, we considered 5 different commonly used RD algorithms GeneConv [3], MaxChi [5], Chimaera [4], Siscan [1] and 3Seq [2]. These algorithms employ different strategies to detect crossovers [4]. GeneConv is a sequence comparison based method that looks for high identity segments in a pair of sequences that exhibit a few variations in other sequences [3]. MaxChi performs pair-wise comparisons at each position by counting the number of differences to the right and to the left of that position. Using a 2×2 matrix of these values, it calculates chi-squared values at each position and proposes positions with maximum chi-squared values as potential recombination sites [5]. Chimaera compares triplets instead of a pair of sequences in which each sequence is considered a potential recombinant. Based on the positional chi-squared values calculated from differences to the right and to the left, the position with a maximum chi-squared value is considered a potential break point [4]. SiScan compares multiple sequences using a window-based approach by randomizing positions within a window and calculating z-scores [1]. 3Seq works on the principle of identifying signals that are less likely to occur under a clonal reproduction model [2]. REDHORSE is different from these RD algorithms in the following ways: 1) REDHORSE is not just a RD algorithm but a complete pipeline that extracts genetic markers from NGS data to construct MSAs. These MSAs are then used as inputs to existing RD algorithms [2-5] 2) The RD algorithm in REDHORSE uses markers throughout the genome to find crossovers. In contrast, other algorithms were designed for lower resolution data such as from RFLP [7] and microarrays [8] and their applicability for NGS data has not been thoroughly tested. Most of these algorithms could not handle markers from all the chromosomes for our NGS data [9]. 3) REDHORSE compares hybrids against only the parental lines to determine the break points. The other RD algorithms perform all-to-all comparisons in sets of twos or threes to determine hybrid parental compositions. 4) REDHORSE uses physical position information to determine if any two break points are within 5000 bp and it categorizes them as double crossovers (DCs). This is not possible using other RD algorithms as MSA files do not include physical position information. 5) The RD algorithm in REDHORSE uses a sliding window, similar to other RD algorithms, to detect potential break points. However, REDHORSE employs a second step where the profile of the hybrid before and after a DC is checked to see if there is a conventional crossover (CC) after DC. 6) Since the inputs to other RD algorithms are MSAs that lack position information, the recombination positions detected by these algorithms need to be manually mapped back to their true genomic positions. REDHORSE on the other hand directly reports the true genomic positions of recombination based on a “merged allele file” containing genomic position information.

REDHORSE is a portable, platform independent, Java-based utility that is compatible with a variety of alignment algorithms. It takes alignments to the genome as input in sequence alignment map (SAM) or binary alignment map (BAM) format [10]. Using a custom single nucleotide variant (SNV) caller and associated utilities, it identifies loci where both the parental strains differ. Based on these loci as markers and using allele information generated using REDHORSE, it builds a “merged allele file” that contains physical marker positions as well as nucleotide composition of all the samples at those markers. This “merged allele file” serves as input to the RD algorithm in REDHORSE that detects crossovers. Further, it converts the “merged allele file” to a MSA file that can be directly used as input to other RD programs. This facilitates a direct comparison of results from RD program in REDHORSE with other software programs that identify recombinations. Therefore, REDHORSE can not only be used independently as a tool to generate MSAs that are inputs to other RD algorithms, but also be independently used for RD. REDHORSE can be run on various operating systems equipped with Java version 1.5 or higher and requires less than 2GB of RAM for smaller genomes (<64 Mb) and may require higher RAM based on the size of the genome. It can be downloaded from http://redhorse.sourceforge.net/ and can be directly used with no installation required.

## Availability and requirements

### Manual

REDHORSE manual pages are accessible through sourceforge at http://redhorse.sourceforge.net/. The analytical pipeline is available through the link “Analytical Pipeline”.

#### Software package

The java archive file can be downloaded from http://sourceforge.net/projects/redhorse/files/release%201.0/Code/.

#### Data download

The NGS data employed in this manuscript and the simulated data can be downloaded from http://sourceforge.net/projects/redhorse/files/release%201.0/data/.

#### Requirements

Java version 1.5 or later.

## Implementation

### Analytical pipeline

**Figure 1:**
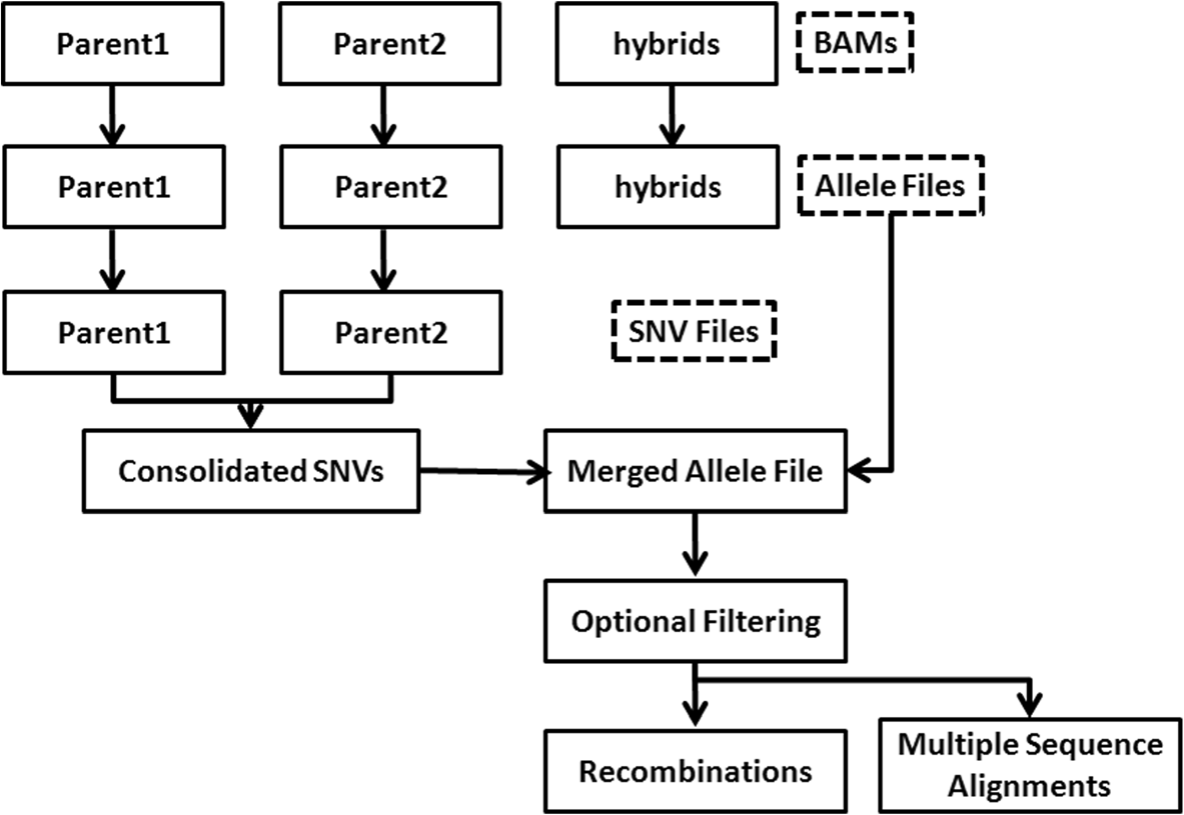
**Pipeline to find recombinations using REDHORSE.** REDHORSE accepts sorted alignments of “reads” to the genome (SAM/BAM) as input. REDHORSE extracts base composition at each genomic position from the BAM files using user defined parameters and compares this information with the reference genome to extract single nucleotide variations (SNVs). It consolidates the parental SNVs and retains markers where both parents are different while filtering out markers where both parents are same but different from the reference genome. It extracts the nucleotide information from all the samples at these marker positions and generates a “merged allele file” that includes physical genome positions and nucleotide information from each sample. And finally, it uses this “merged allele file” to extract CCs and DCs. It also converts the “merged allele file” to MSA format to be used as input to other RD algorithms. Optional filtering steps are not listed in this figure, see Methods.

This section outlines the analytical pipeline (Figure 1) of REDHORSE that is used to convert the genomic alignments of the “reads” to MSAs that can be used as input to RD algorithms. The detailed steps involved in the design of RD algorithm of REDHORSE are also presented.

### Alignment

REDHORSE uses alignments of NGS reads to the genome of interest as input. A variety of alignment algorithms have been designed for aligning the reads to the genome [11-13]. We performed the alignments of short reads to the ME49 v8.0 genome (Toxodb- http://www.toxodb.org//) using CLC genomics workbench (CLC Bio V6.0.2) using the parameter set: mismatch cost of 3, insertion cost of 3, deletion cost of 3, length fraction 0.9, similarity fraction 0.8 and by using global alignment. The aligned reads were sorted by genomic position and the sorted BAM file was used as input to REDHORSE.

### Find nucleotide composition at each genomic position

The first step in the analytical pipeline was to parse the bam file to generate an “allele list file” that contained nucleotide composition of the sample at each genomic position. This file is a tab-delimited file with columns 1) genomic position, 2) nucleotide composition, 3) read coverage, 4,5,6,7) nucleotide frequencies of A,T,C,G at that position, 8) number of forward reads, 9) number of reverse reads and 10) average mapping quality. The user can choose the minimum allele frequency to call the allele composition. For diploid organisms, heterozygous allele frequencies are close to 50% and for triploid organism, minor allele occurs with ~33% frequency and major allele occurs with ~66% frequency and so on. The frequency threshold used in allele generation therefore must be chosen appropriately. Since we were dealing with a haploid genome, and since heterozygous sites were unexpected, we used a minimum allele frequency threshold of 80% to call the alleles. For haploid genomes, any minor alleles occurring with less than 20% frequency represent noise that arose because of sequencing, genome composition, mis-alignment, population based artifacts and other experimental artifacts. If at any given position there was no allele that meets this frequency criterion, it was reported as missing data (−). We filtered out background noise by ignoring all the regions with coverage less than 5 and filtered out noise due to repeats by ignoring regions with coverage greater than 2x of the average chromosomal coverage.

### Find single nucleotide variations (SNVs)

REDHORSE implements a custom built algorithm to identify SNVs using the allele file and the reference fasta file. We designed a SNV caller whose parameters can be adjusted for haploid, diploid or aneuploid genomes. This was done at the allele generation step where minor allele frequency can be defined. The SNV caller compares the allele information at each genomic position from the sample against the reference genome and finds the loci that are different. The SNV caller does not make assumptions regarding the ploidy of the data but rather makes use of the allele information that is generated based on the user defined thresholds. For paired-end data, it ensures that reads are in both the orientations where SNVs are called. We called SNVs from the regions where there were at least 25% of reads (user defined parameter) in either orientation. The loci where there were more than 3 SNVs in a 7 bp window (user defined parameters) were filtered out. These two filters are used to avoid calling SNVs falsely from regions where there are insertions or deletions. The program outputs standard information such as chromosome, position, reference allele and alternate allele in tab-delimited or standard variant call format (vcf).

### Consolidate the SNVs

Typically, the reference genome used is the assembly of one of the parents or an assembly that is close to both the parental strains that are being studied. The SNVs found using the parental strains represent the loci where each of the parents was different from the reference genome. The loci of interest were the loci where both parents were different from each other. It was possible that both the parental strains were different from the reference genome but were similar to each other (typical of reference assembly errors). REDHORSE retains loci where both parental strains are different and filters out loci where both parents were the same, but were different from the reference genome.

### Generate “merged allele file”

The detection of recombinations in the hybrids using existing RD algorithms requires generating MSAs. REDHORSE takes the consolidated SNV file and finds the allele composition from all the hybrids and parents at the variant loci. This master file is called the “merged allele file” and it contains the allele composition of the parents as well as the hybrids and their actual marker positions in the genome. This file can be used to infer the CCs and DCs in each hybrid after undergoing multiple optional filtering stages: 1) The loci where either of the parents have missing data were filtered out as it is not possible to adequately assess if both parents were the same at those loci and also they do not allow comparison between hybrids and parents. 2) The loci which have 30% (user defined parameter) or more missing data from the hybrids were filtered out as they represent data from unreliable regions such as repeats and 3) The loci where hybrids have more than 2 alleles (user defined parameter), also called multi-allelic loci were filtered out as they represent sequencing errors or population based artifacts.

### Algorithm to find conventional recombinations and double crossovers

**Figure 2:**
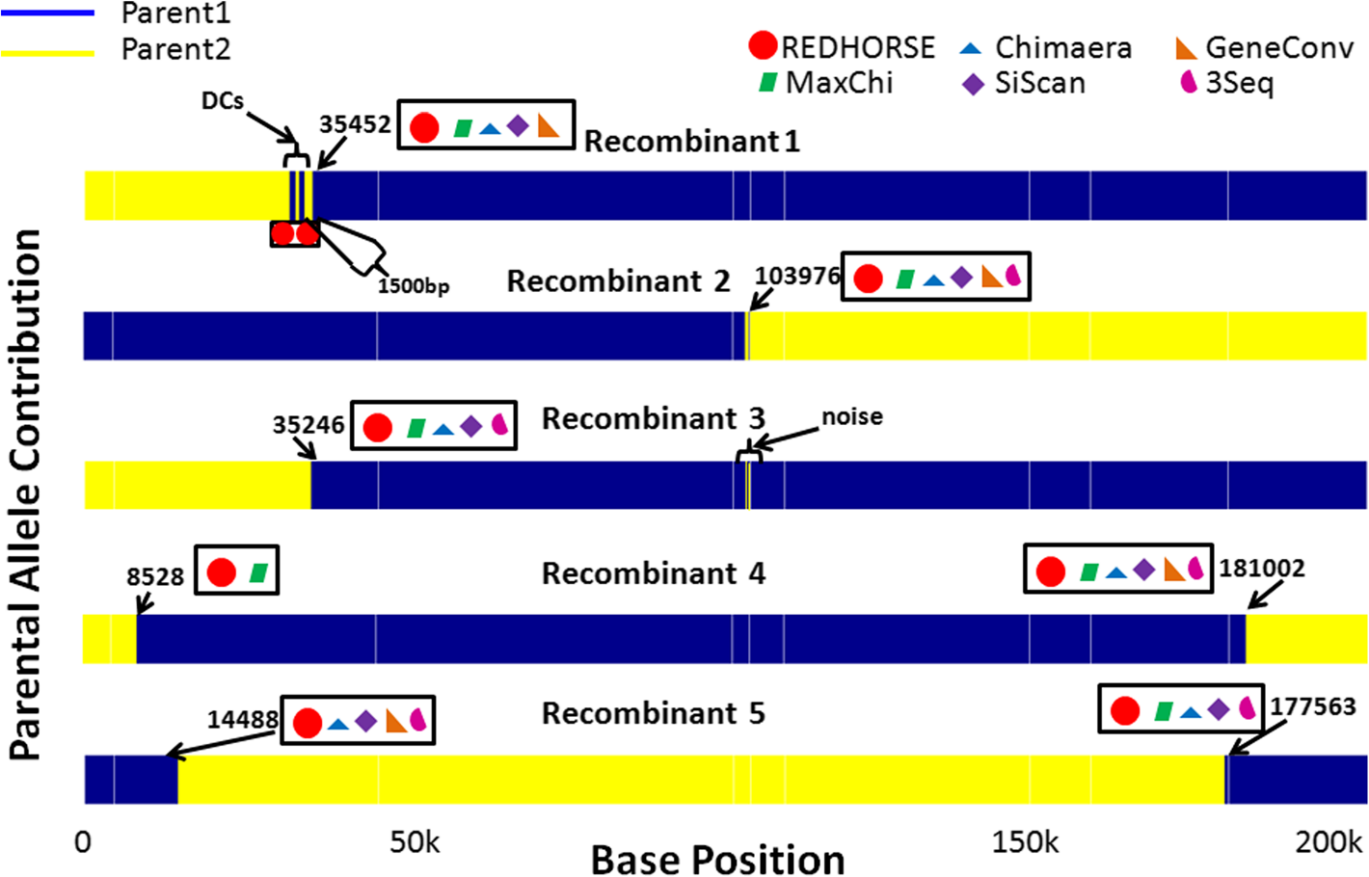
**Visual depiction of simulated dataset containing DCs and CCs.** The parental profiles and the hybrid profiles were drawn using markers as described in the section “Analytical Pipeline”. Each marker was represented using a vertical bar of height 1 unit and the bars for parent 1 were given a dark blue color and those for parent 2 were given a yellow color. The hybrids were drawn based on their genomic composition inherited from each parent. The regions where there were no markers showed up as white regions. Recombinant 1 had one conventional crossover and two DCs placed next to it (<1500 bp). Recombinants 2 and 3 had one conventional recombination each but recombinant 3 had noise introduced into it to replicate experimental artifacts. Recombinants 4 and 5 represented recombinants with multiple recombinations but having different profiles respectively. The break points detected by individual algorithms were indicated next to the break points in color-coded shapes respectively.

The “merged allele file” facilitates comparison of hybrids against the parents but also allows detection of DCs based on the genomic positions of the markers. The RD algorithm in REDHORSE employs an iterative procedure to find recombinations. The steps involved are as follows:

Step 1: Using a window size of 10 and a step size of 1, REDHORSE scans each hybrid by comparing them against the parental strains (Additional file 1: Figure S1(a)). All loci that show a switch from one parent to the other supported by at least 6 markers (> half the window size) were tagged as putative break points
The break points that were ≤ 5000 bp (user defined value) and separated by at least 6 markers (user defined value) were deemed putative DCs (Additional file 1: Figure S1(b)) and were filtered out from putative CCs detected in Step 1. The choice of 5000 bp was based on our observations from the real data (see companion manuscript [9]).
Step 3: The break points that were ≤ 5000 bp (user defined value) and were separated by less than 6 markers or break points that were less than 250 bp apart were deemed noise and removed from putative crossovers detected in step 2. We determined them to be noise by sequencing some of those regions (see companion manuscript [9]).
Step 4: The putative DC boundaries sometimes overlapped with the CC boundaries. These usually occurred at loci where there were multiple DCs. The boundary crossover points may or may not be CCs so they were putatively included in the CC list to be verified next.
Step 5: The putative CCs defined in steps 2–4 went through a second evaluation stage where boundaries included in step 4 were evaluated by comparing against other break points. The break points that indicate DCs that had overlapping boundaries with CCs (see Figure 2-Recombinant 1) were adjusted to report both DCs and CCs appropriately. If the profile immediately before DCs and immediately after DCs are the same, only the DCs are reported and if the profiles are different (see Figure 2 Recombinant 1), then both the DCs and the adjacent CC are reported.

### Datasets

This section describes the datasets employed in this manuscript for demonstrating the utility of REDHORSE.

### Simulated dataset

**Figure 3:**
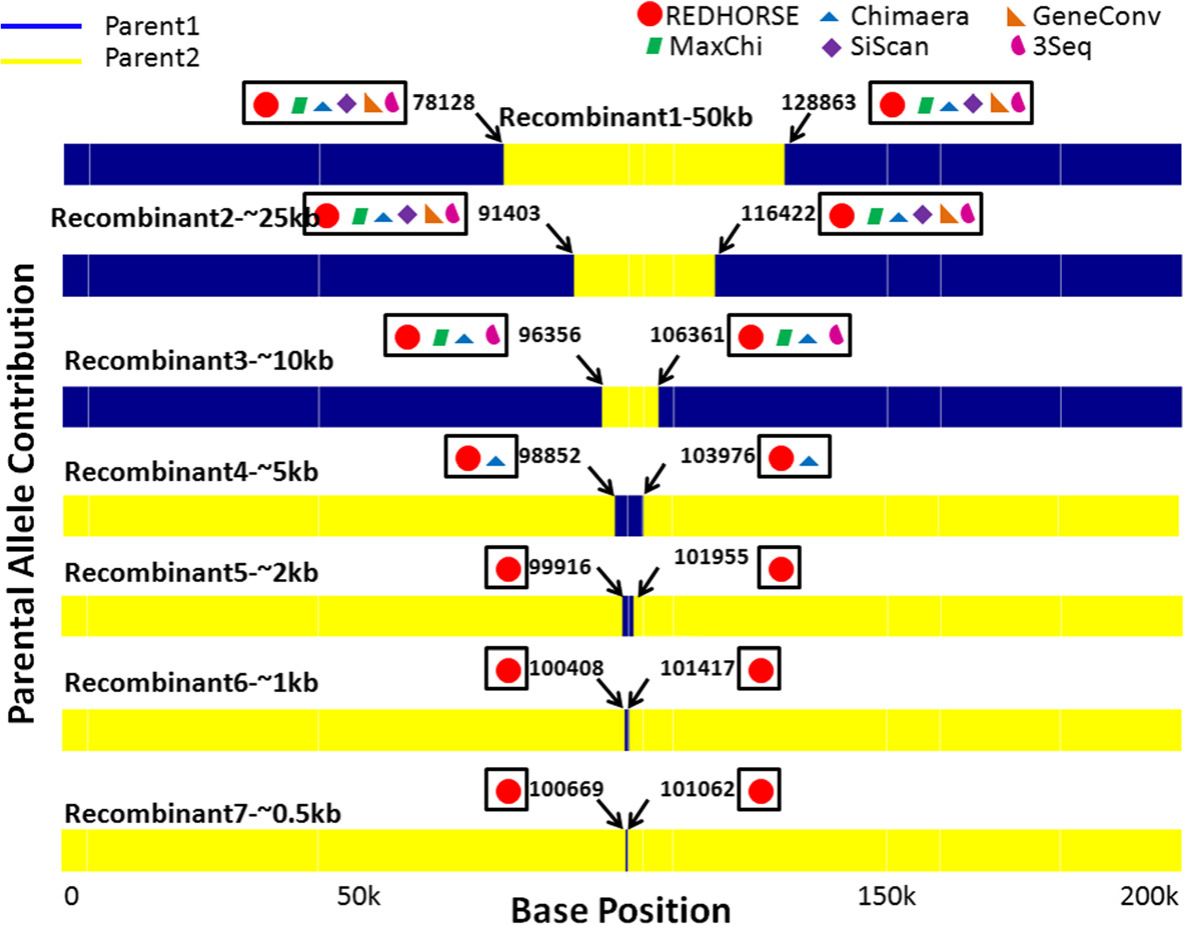
**Visual depiction of a simulated dataset containing two break points separated by 50 kb-0.5 kb.** The parental profiles and the hybrid profiles were drawn respectively similar to Figure 2. DCs of various sizes were introduced to generate 7 recombinants. Break points separated by greater than 25 kb were detected by all the RD algorithms. Break points separated by greater than 5 kb and less than 25 kb were detected by only a few RD algorithms. Break points less than 5 kb typical of DCs were detected only by REDHORSE.

The simulated dataset1 was created by generating a random reference sequence of 200 kb, from which two parental strains were derived. The first parental strain was generated by introducing ~2% of SNVs randomly to the reference sequence to mimic a parental strain that was different from the reference genome. The second parental strain was generated by introducing ~0.2% of SNVs to the reference genome randomly to mimic a strain that was close to the reference genome. SNV differences between the reference and two parental strains were assigned independently. Using the above criteria, a total of 4337 SNVs were randomly introduced into two parental strains. For evaluating the performance of RD algorithms to detect CCs, a total of 5 recombinants, 3 containing single crossovers and 2 containing 2 crossovers were generated by randomly choosing the break points. Two DCs mimicking gene conversions were placed in proximity (<1500 bp-see Figure 2) to the CC on recombinant 1. These DCs evaluate how individual RD algorithms interpret the common boundaries between the two DCs and between the second DC and CC next to it. A few noisy impulses were introduced on recombinant 3 to determine if they will be mistaken for DCs by the RD algorithms. Noisy impulses were defined as two break points separated by less than 250 bp (and/or) one or two boundaries that were not supported by 6 markers indicating a switch. The parental sequences and hybrid sequences were fed to the wgsim package [14] and simulated NGS reads were generated. A total of 60000 reads (30000 paired end reads), each of length 50 bp separated by an average length of 50 bp were generated for parental and hybrid sequences respectively with mutation rate of 0.005 and indel fraction of 0.1. This accounts for a modest average read coverage of 30× across 200 kb genome. A second simulated dataset was generated for evaluating the performance of RD algorithms to detect DCs. A total of 7 hybrids were generated with two break points separated by 50 kb-0.5 kb respectively (see Figure 3). This was done by copying the parental profiles from the “merged allele file” generated using simulated dataset1 and generating hybrids by manually introducing two break points at various distances (50 kb-0.5 kb) in each hybrid respectively.

### NGS dataset

*Toxoplasma gondiii* is a haploid parasite that causes opportunistic disease in humans. Previous genetic mapping studies have relied on RFLP markers [7] or SNVs called from microarray hybridizations [8]. However, such methods were limited by being laborious or requiring that the array probes were capable of defining polymorphism between strains. As genetic crosses were done with a wider variety of genetic strains, this became problematic to scale. In addition, with the advent of NGS, it became much easier to generate the allele data. Here we sought to implement techniques using NGS data to generate new genetic maps. Although a complete summary of these findings appears separately [9], here we described the development of software tools to analyze genetic crosses between haploid genomes that is illustrated using chr VIII of *T. gondii*.

## Results

### Demonstration of the utility of REDHORSE

The utility of REDHORSE was demonstrated using both the simulated data and NGS data. The simulated dataset allows one to introduce different features into the recombinants and to study how individual algorithms perform. The dataset from chromosome VIII of *Toxoplasma gondii* (described in an accompanying paper, [9]) allowed us to demonstrate the pipeline using NGS data.

### Analysis using simulated datasets

The simulated datasets were used to evaluate the performance of RD algorithms based on their ability to detect DCs as well as CCs. Since it was a simulated dataset, the break points were known apriori and could be compared against the break points found using the RD algorithms. The simulated reads generated by the wgsim package (see section “Datasets”) were aligned to the simulated reference genome using novoalign [13]. We used a different alignment algorithm from the algorithm used for the real dataset to show the compatibility of REDHORSE with different aligners. The REDHORSE pipeline was followed to generate a “merged allele file” and to find recombinations. The “merged allele file” was converted to a MSA file and was used as input to other RD algorithms.

#### Conventional crossover detection using simulated data one

A desirable RD algorithm is the one that can detect authentic recombinations, whether simple or complex, whilst ignoring the experimental artifacts. To simulate the ability to differentiate DC boundaries from CC boundaries, we strategically placed DCs next to each other and within 1500 bp distance to a CC. This arrangement was designed to test RD algorithms for their ability to identify individual DCs and distinguish them from CCs. We also introduced noise (see “Datasets”) into recombinant 3 to test the RD algorithms ability to differentiate noise from true DCs.

##### Analysis using REDHORSE

There are seven break points corresponding to CCs in all the recombinants and four break points corresponding to DCs on recombinant 1. All the seven CC break points were detected using the RD algorithm in REDHORSE (Figure 2). REDHORSE was also able to identify boundaries of two DCs introduced into recombinant 1 and adjusted the boundaries as expected. REDHORSE not only detected the CC breakpoint on the hybrid 1 at 35452 but also detected the DC next to it and adjusted its boundaries (33421–35451), 35452 being the common break point for the DC as well as CC. It was able to ignore the noise introduced on hybrid 3 and did not falsely call it a DC.

##### Analysis using existing RD algorithms

Since the existing RD algorithms employed in this manuscript employ MSAs, we converted the “merged allele file” into a MSA fasta file and used this file as input to other RD algorithms. We compared 3Seq [2], Chimaera [4], siScan [1], GeneConv [3] and maxChi [5], which are some of the most popular and easy to use software programs. The results indicate that these algorithms were able to detect a subset of CCs (Figure 2). In particular, the other RD algorithms had difficulty finding crossover at the beginning of the chromosome (bp.8528) on recombinant 4. None of these algorithms were able to detect the DCs.

#### Double crossover detection using simulated data two

We were intrigued by the fact that none of the existing RD algorithms detected any DCs. To probe into this further, we generated a second simulated dataset containing 7 recombinants by introducing two break points separated by a distance of 50 kb-0.5 kb. We did this by manually introducing break points in the “merged allele file” (see “Datasets”). The plots (Figure 3) showing the allele composition of the parents as well as recombinants were drawn based on the SNV markers.

##### Analysis using REDHORSE

The seven recombinants contain two break points each separated by 50 kb-0.5 kb. The break points separated by greater than 5 kb (recombinants 1–4) were all detected by the CC detection algorithm in REDHORSE. The break points separated by less than 5 kb (recombinants 5–7) were detected by the DC detection algorithm in REDHORSE (Figure 3).

##### Analysis using existing RD algorithms

Similar to our simulated dataset one, we converted the “merged allele file” into a MSA fasta file and used this file to find recombinations by several programs. The results indicate that one or more of these algorithms were able to detect break points greater than 5 kb but they were unable to detect breakpoints separated by less than 5 kb (Figure 3).

#### Thoughts on RD algorithms using two simulated datasets

REDHORSE was the only algorithm that detected the break points that were separated by <5 kb, typically indicative of DCs. When the DCs were placed close to a CC, REDHORSE identified and adjusted the boundaries of DCs and CCs. Some of the other RD algorithms were able to detect only the CC nearby but not the DCs. The noise mimicking DCs on recombinant 3 were ignored by REDHORSE. The other algorithms did not detect DCs nor did they highlight noisy impulses due to their overall lower sensitivity. REDHORSE was able to detect 100% of break points (CCs and DCs) using the simulated datasets where as the other algorithms (MaxChi-56%, Chimaera-56%, SiScan-56%, 3Seq-46% and GeneConv-46%) only had limited success in detecting these break points.

### Analysis using chromosome VIII of the NGS Dataset

A representative chromosome VIII from the two parental strains (ME49 and VAND) of *Toxoplasma gondii* and three hybrids (see section Datasets) from a recently described genetic cross [9] were used to demonstrate the utility of REDHORSE on NGS datasets. By showing its utility on a representative data, we intend to demonstrate that our pipeline can be applied to frameworks involving similar NGS data.

#### SNV calling and consolidation

The genomic alignments of short reads (bam files) were used as input to REDHORSE to generate “allele files”. The files corresponding to parental strains were used as input to the SNV caller in REDHORSE to call the SNVs. A total of 130 ME49 SNVs and 59,932 VAND SNVs were detected. Ideally you would expect that the ME49 sequence data would perfectly match the ME49 reference but minute variations can be attributed to events such as errors in assemblies, population based effects and repeat regions. Since we were interested in loci where both parental strains are different from each other, we consolidated the two lists to exclude SNVs that were common to both the parental strains, resulting in a total of 59,953 SNV markers where parental strains were different from each other.

#### “Merged Allele” generation and filtering

The allele files and the markers from the consolidated SNV file were used as input to REDHORSE to generate a “merged allele file”. Multiple filters were applied on the “merged allele file” by removing the loci where either of the parents have missing data, removing multi-alleleic loci and filtering out loci where more than 30% of the samples have missing data, resulting in a total of 57,863 loci.

#### Finding recombinations and double crossovers using REDHORSE

**Figure 4:**
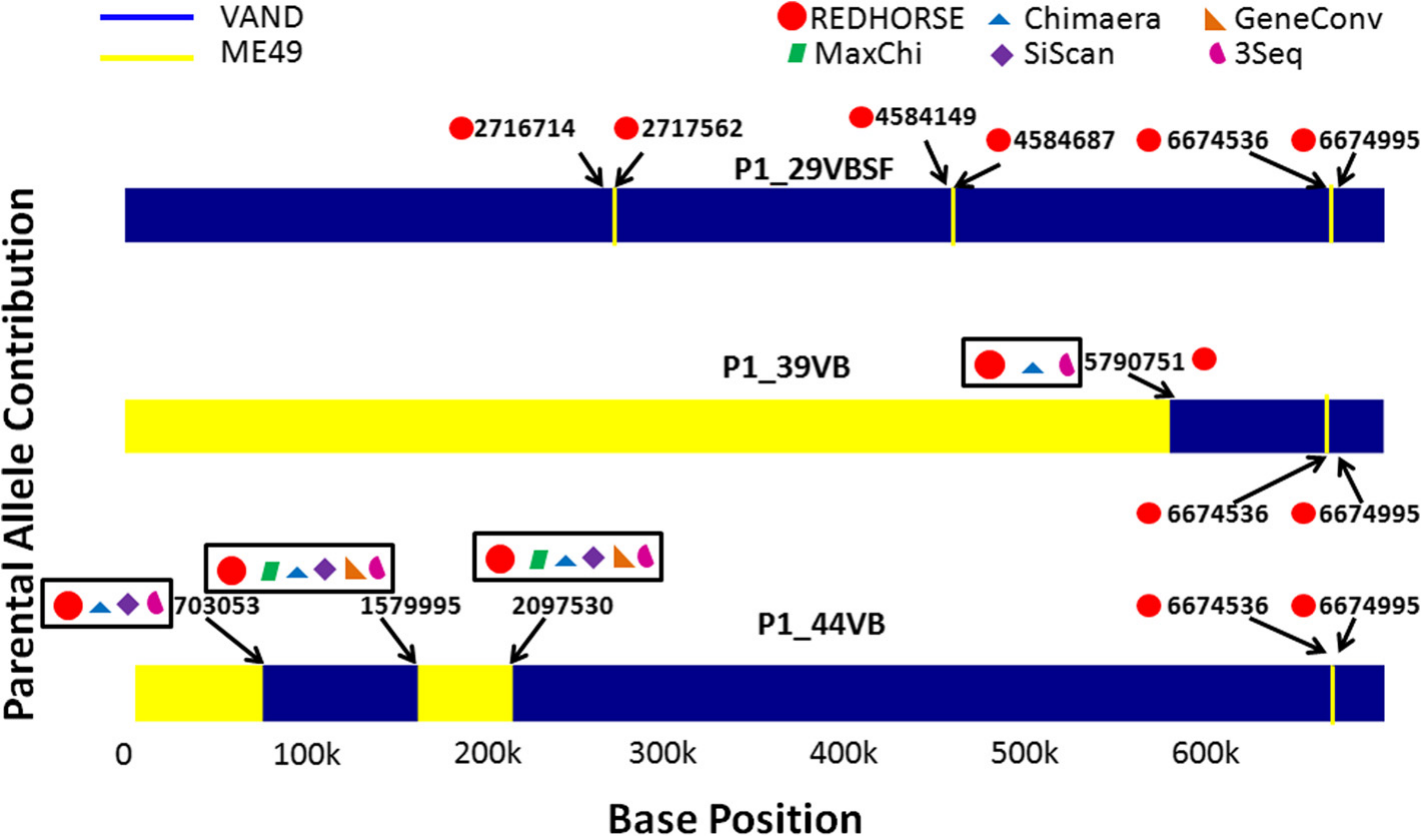
**Visual depiction of NGS data of experimental hybrids using Chromosome VIII of Toxoplasma gondii.** These plots were drawn similar to the simulated datasets by coloring hybrids according to their composition and inheritance from each parent (VAND-dark blue and ME49-yellow). The hybrid P1_29VBSF had no CCs but had three DCs. The hybrid P1_39VB had a CC and a DC. The hybrid P1_44VB had multiple CCs and a DC. The break points detected by individual algorithms were indicated next to the break points in color-coded different shapes respectively.

The “merged allele file” was used as input to REDHORSE to detect CCs as well as DCs. REDHORSE detected CCs in two out of three hybrids, suggesting one of the hybrids did not have any CCs. The profiles of the parents as well as hybrids that were plotted based on their genomic composition show that only two of the samples had CCs (Figure 4). The hybrid P1_39VB had one break point suggesting one CC where as the sample P1_45VB had three break points suggesting three CCs all of which were detected by REDHORSE. The DC detection algorithm in REDHORSE detected three distinct DCs with one DC common across all the three hybrids and the other two that were specific to P1_29VBSF.

#### Finding recombinations and double crossovers using existing RD algorithms

We converted the “merged allele file” to MSA format and used it as input to other RD algorithms. As with our simulated data, we used 3Seq, Chimaera, siScan, GeneConv and maxChi algorithms. These RD algorithms were able to detect a subset of CCs (Figure 4). Similar to results using our simulated data, these RD algorithms were not able to detect any of the DCs.

#### Thoughts on RD algorithms using real NGS data

As observed with our simulated data, the other RD algorithms did not detect DCs from the real data. REDHORSE RD algorithm was the only algorithm that identified all the observable CCs and DCs with 100% accuracy where as the other algorithms (MaxChi-30%, Chimaera-46%, SiScan-43%, 3Seq-46% and GeneConv-30%) had limited success in detecting these break points.

## Discussion

Genetic recombination, single nucleotide polymorphisms and structural variations are the primary processes by which living organisms generate diversity [15]. Various algorithms have been developed to identify recombinations in haploid as well as diploid organisms [1-5,16-20]. The detection of crossovers in haploid genomes involves direct comparison of hybrids against the potential parental strains [1-5] or determining the parental compositions de novo. For the diploid organisms, the problem is complex and is usually addressed by generating genetic linkage maps, estimating recombination frequencies and attributing recombinations to loci based on centimorgan distances [17-21]. Evolving technologies demand efficient pipelines to summarize the data in a format that can be used as input to the RD algorithms. The proposed analytical pipeline extracts meaningful loci that differ among genomes, determines nucleotide composition at those loci and summarizes the data in a format that has a wide range of applications including RD. REDHORSE employs an analytical pipeline that systematically removes noise to generate MSAs. The RD algorithm in REDHORSE employs an iterative scheme and uses the genomic positions to detect both CCs and DCs. REDHORSE was the only algorithm that was able to reliably detect DCs whereas all other RD algorithms had lower sensitivity. We introduced a new file format called a “merged allele file” that contained information about genomic positions as well as sequence alignments from all the samples. The analysis using this file format has added advantages in detecting DCs where distance between two consecutive break points can be calculated using the true genomic positions. REDHORSE can be independently used for SNV detection, allele extraction, and generation of MSA files. REDHORSE is written in Java and therefore is platform independent with no installation required. The HTML manual pages contain clear instructions along with commands and snippets of the output that make even novice users to follow with ease.

## Conclusion

We developed an efficient Java-based toolset called REDHORSE and demonstrated its use as an efficient analytical pipeline to convert NGS alignments to MSAs and to extract recombination points. The use of NGS data provides a dense set of markers throughout the genome to study events such as recombinations with high accuracy. REDHORSE serves as an interface between the existing tools and the NGS technologies for RD. We developed a RD algorithm that handles high volume of data and makes use of marker position information to accurately detect break points. The utility of REDHORSE is demonstrated using simulated datasets as well as NGS data. The simulated datasets mimic a real world scenario of recombination between two independent parental lines where the crossover loci are known and can be used to compare against the break points detected using individual RD algorithms. We plotted the profiles of parental lines and hybrids respectively using the SNV markers and using the composition of the parents as reference. The break points detected using REDHORSE as well as the other existing RD algorithms are visually depicted using these plots. The ability of REDHORSE to extract hidden patterns such as DCs, which may be biologically relevant, while filtering out artifacts and its ability to work with NGS sized data make it favorable for RD. REDHORSE is a comprehensive suite for RD in haploid genomes that can be run on any operating system supporting Java 1.5 or higher. It is memory efficient and can be run on machines with 2GB of RAM or higher.

## Additional file

Additional file 1: Figure S1. Illustration of terminology used in the manuscript. (a) Window, marker and break point illustrated. Window is the moving window of size 10 markers and of step size one marker employed by REDHORSE to scan for potential break points. Markers are the loci (regions of a genome) where both parental lines are different from each other. Break point is the locus where at least 3 markers from each parent are observed. (b) A double crossover is a locus where two break points are separated by less than 5000bp and are separated by at least 6 markers. (c) Noise is defined as a brief switch a hybrid makes from one parent to the other or has a profile different from both the parents. Noise is also a region with extremely low coverage or extremely high coverage typical of repeats. Highlighted markers are not next to each other physically but they are two immediate SNVs separated by a certain distance in bp.

## Abbreviations

RD: Recombination detection
DC: Double crossover
CC: Conventional crossover
NGS: Next-generation sequencing
MSA: Multiple sequence alignment; SAM: Sequence alignment map
BAM: Binary alignment map
SNV: Single nucleotide variations

## References

[1] Armstrong MJ, Gibbs AJ (2000). Sister-Scanning: A Monte Carlo procedure for assessing signals in recombinant sequences. Bioinformatics.

[2] Boni MF, Posada D, Feldman MW (2007). An exact non-parametric method for inferring mosaic structure in sequence triplets. Genetics.

[3] Padidam M, Sawyer M, Fauquet CM (1999). Possible emergence of new gemini viruses by frequent recombination. Virology.

[4] Posada D, Crandall KA (2001). Evaluation of methods for detecting recombination from DNA sequences: Computer simulations. Proc Natl Acad Sci.

[5] Smith JM (1992). Analyzing the mosaic structure of genes. J Mol Evol.

[6] Wang J, Fan HC, Behr B, Quake SR (2012). Genome-wide single-cell analysis of recombination activity and de novo mutation rates in human sperm. Cell.

[7] Khan A, Taylor S, Su C, Mackey AK, Boyle J, Cole R, Glover D, Tang K, Paulsen IT, Berriman M (2005). Composite genome map and recombination parameters derived from three archetypal lineages of *Toxoplasma gondii*. Nucleic Acids Res.

[8] Behnke MS, Fentress SJ, Mashayekhi M, Li LL, Taylor GA, Sibley LD (2012). The polymorphic pseudokinase ROP5 controls virulence in *Toxoplasma gondii* by regulating the active kinase ROP18. PLoS Pathog.

[9] Khan A, Shaik JS, Behnke M, Wang Q, Dubey JP, Rosenthal BM (2014). Analysis of recombination by NextGen sequencing reveals frequent small crossovers as a major source of generating diversity between exotic and clonal strains of Toxoplasma gondii. BMC genomics.

[10] Li H, Handsaker B, Wysoker A, Fennell T, Ruan J, Homer N, Marth G, Abecasis G, Durbin R, subgroup gpdp (2009). The Sequence Alignment/Map format and SAMtools. Bioinformatics.

[11] Li H, Durbin R (2009). Fast and accurate short read alignment with Burrows-Wheeler Transform. Bioinformatics.

[12] Langmead B, Trapnell C, Pop M, Salzberg SL (2009). Ultrafast and memory-efficient alignment of short DNA sequences to the human genome. Genome Biol.

[13] Novocraft.com. Novoalign short read mapper. http://www.novocraft.com/main/downloadpage.php; 2013.

[14] Li H. wgsim - Read simulator for next generation sequencing. http://github.com/lh3/wgsim 2013.

[15] Ajzenberg D, Banuls AL, Su C, Dumetre A, Demar M, Carme B, Darde ML (2004). Genetic diversity, clonality and sexuality in Toxoplasma gondii. Int J Parasitol.

[16] Amos C, Zhu DK, Boerwinkle E (1996). Assessing genetic linkage and association with robust components of variance approaches. Ann Hum Genet.

[17] Browning SR, Browning BR (2007). Rapid and accurate haplotype phasing and missing-data inference for whole-genome association studies by use of localized haplotye clustering. Am J Hum Genet.

[18] Jombart T (2008). adegenet: a R package for the multivariate analysis of genetic markers. Bioinformatics.

[19] Lander E, Green P (1987). Construction of multilocus genetic linkage maps in humans. Proc Natl Acad Sci U S A.

[20] McKeique PM (2005). Prospects of admixture mapping of complex traits. Am J Hum Genet.

[21] Broman KW, Wu H, Sen S, Churchill GA (2003). R/qtl: QTL mapping in experimental crosses. Bioinformatics.

