# Supplementary figures and images for: REDHORSE-REcombination and Double crossover detection in Haploid Organisms using next-geneRation SEquencing data
Source: BMC Genomics. 2015 Feb 26;16(1):133. doi: 10.1186/s12864-015-1309-7 (PMC4348101; doi:10.1186/s12864-015-1309-7)

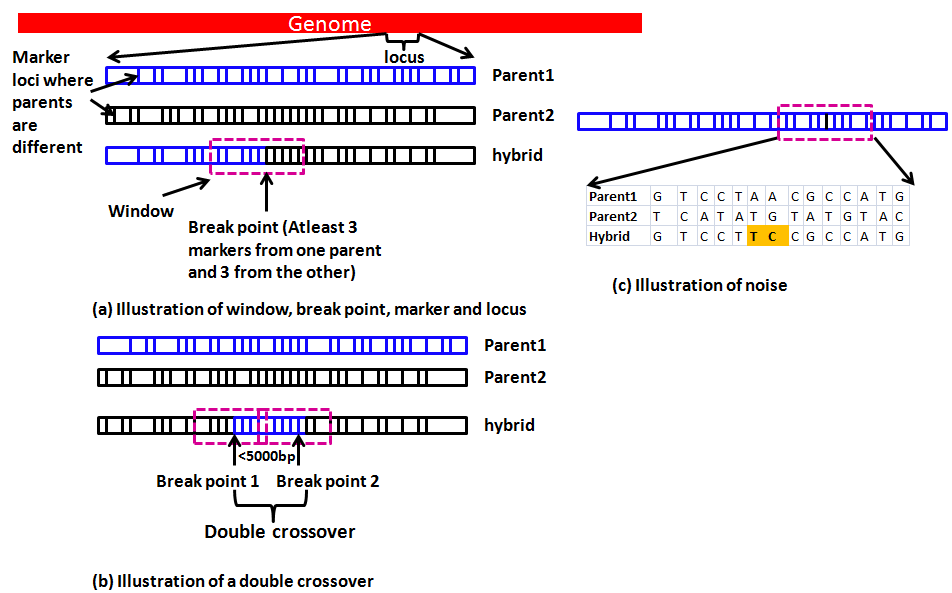

Supplement: Additional file 1: Figure S1. — Illustration of terminology used in the manuscript. (a) Window, marker and break point illustrated. Window is the moving window of size 10 markers and of step size one marker employed by REDHORSE to scan for potential break points. Markers are the loci (regions of a genome) where both parental lines are different from each other. Break point is the locus where at least 3 markers from each parent are observed. (b) A double crossover is a locus where two break points are separated by less than 5000bp and are separated by at least 6 markers. (c) Noise is defined as a brief switch a hybrid makes from one parent to the other or has a profile different from both the parents. Noise is also a region with extremely low coverage or extremely high coverage typical of repeats. Highlighted markers are not next to each other physically but they are two immediate SNVs separated by a certain distance in bp. [file 12864_2015_1309_MOESM1_ESM.png]
